# PROTOCOL: Adult skills development and training in high‐income countries: A Campbell evidence and gap map

**DOI:** 10.1002/cl2.1126

**Published:** 2020-10-28

**Authors:** Vivian Welch, Christine Mathew, Luciana M. Marins, Elizabeth T. Ghogomu, Sierra Dowling, Salman Abdisalam, Mohamad T. Madani, Emma Murphy, Kisanet Kebedom, Jennifer Ogborogu, Kelly Gallagher‐Mackay

**Affiliations:** ^1^ Bruyere Research Institute Ottawa Ontario Canada; ^2^ Future Skills Centre—Centre de Compétences futures Toronto Ontario Canada

## 1 BACKGROUND

### 1.1 The problem, condition, or issue

Human capital is a chief driving force of economic development. The Organization for Economic Co‐operation and Development (OECD) estimated that approximately 9% of current jobs within OECD member states are threatened with automation and digitalization—all significant successes and advances in artificial intelligence, robotics, and computer science (Arntz et al., 2016). With such global changes and forecasts, in the labor market, there are ever‐evolving demands on employees and employers to gain or update new and/or different skill sets and competencies. Governments and policy‐makers at various levels have shifted towards investments and surveillance of various types of job training and skills acquisitions (OECD, 2009). Active labor market programs help to bring individuals into employment, keep workers employed, optimize productivity and earnings through various means (Brown & Koettl, 2012).

Further to the threat of automation and digitalization, international organizations are monitoring and estimating labor market trends considering COVID‐19 and its impact on the various economies worldwide. As of September 22nd, 2020, there have been over 31 million cases and over 960,000 deaths associated with COVID‐19 (John Hopkins University, 2020) with numbers still increasing daily. The consequences of this pandemic will include a significant disruption and impact on global unemployment and underemployment rates, implications for the quality of work available with reduced income, and working poverty, and worsening social inequalities and inequities as less access to social protection may also impact the quality of work done by employees (International Labor Organization, 2020). Furthermore, these critical implications can contribute to a global economic recession (UN Conference for Trade and Development, 2020). COVID‐19 has led to increased demand for some occupations and shortage in specific skills as well as social displacement of workers, for example, some staff have been redeployed to new posts and newly trained healthcare professionals were introduced earlier than expected into needed posts (such as screening for COVID‐19). Evidence on effects of labor market programs to reduce unemployment from previous economic crises may be applicable to the COVID‐19 recovery plan. A key challenge will be to reduce unemployment by skills development and training, whether through upskilling to remain acquainted with and ensure skills match present labor market needs, or reskilling. The UN suggests that skills development programs may be a part of a policy response to COVID‐19. Skills development and training is important by virtue of the outcomes they can produce at both the system, employer, and individual levels.

### 1.2 The intervention

**Figure 1:**
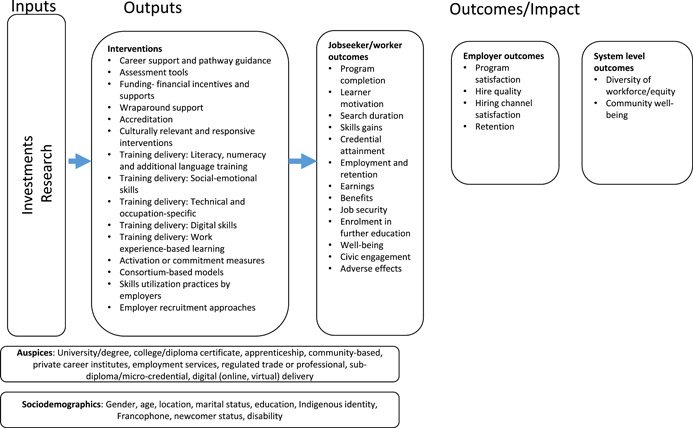
Conceptual framework of interventions and outcomes

Different interventions involving formal education, informal training, and other learning activities can contribute to technical and soft adult skills development necessary in the labor market. The types of interventions include career support, pathway guidance, assessment, funding, wraparound support (e.g., childcare, coaching), accreditation, training delivery (in‐person, digital, practitioner‐led training, by public or private providers; Orlik, 2018). These interventions are often provided in various combinations and may be geared toward paid employment or self‐employment. They can lead to an impact at the participant level (e.g., skills, employment and retention, earnings), and at the level of the employer (e.g., retention, hire quality) and the system (e.g., diversity of workforce/equity, community well‐being). Although many programs have a positive impact on participants, employers and labor market systems, some negative impacts such as reduced wages and displacement have occurred as a result of imbalance in labor demand and supply (Brown & Koettl, 2012; Crépon et al., 2013). See the conceptual framework of interventions and outcomes we developed in consultation with stakeholders who have expertise in skills development and policy (Figure 1).

### 1.3 Why it is important to develop the evidence and gap map (EGM)

An evidence and gap map EGM is a decision making and research prioritization tool used to identify areas of evidence as well as gaps in research to inform social policy, program, and research priorities (Snilstveit et al., 2013). The roles of artificial intelligence and other disruptive technologies in economies have gained prominence among governmental and legislative policy agendas (Jansen et al., 2020). This EGM is aligned with the priorities of the Future Skills Centre (FSC), a pan‐Canadian initiative, and who will be the map's primary user. FSC's mandate is to connect ideas and innovations generated across Canada so that employees and employers may thrive in the labor market, and to ensure that economies at the local, regional, and national levels flourish. Working with their partners to inform and support local approaches to skills development and employment training in ever‐changing economies, FSC will use this rapid and evidence gap map to direct their future research agenda, which is time‐sensitive due to the social and economic consequences of COVID‐19.

### 1.4 Existing EGMs and/or relevant systematic reviews

Similar EGMs exists with a focus on higher levels of education and skills (Winters, 2018), funding for adult skills development (Gloster et al., 2016) and youth skill development in low‐middle income countries (Rankin et al., 2015). Two systematic reviews have also been conducted one on vocational and business training and women's labor market outcomes in low‐and middle‐income countries (Chinen et al., 2017) and the other on youth employment programs and labor market outcomes in high‐ and low‐middle income countries (Kluve et al., 2019).

Since there are existing EGMs for skills development in low‐ and middle‐income countries and the setting for skills development is different in low‐resource settings, we decided to focus on high‐income country evidence for this EGM.

## 2 OBJECTIVES

The objective of this EGM is to identify primary studies and systematic reviews on the effects of adult skills development and training on outcomes for the jobseeker/employee, employer and labor market systems in high income countries.

## 3 METHODOLOGY

### 3.1 Defining EGMs

We will adapt EGM methods from key methods papers (Bragge et al., 2011; Lum et al., 2011; Snilstveit et al., 2013, 2016) and will adopt the suggested five‐stage process:

1. Define a framework.
2. Identify the available evidence.
3. Appraise the quality of the evidence.
4. Extract, code, and summarize the data that relate to the objectives.
5. Visualization and presentation of the findings in a user‐friendly manner.

We will use the Campbell Collaboration mapping tool developed by the EPPI‐Centre (Digital Solution Foundry & EPPI‐Centre, 2020) to generate and display identified studies using the framework described below.

### 3.2 Framework development and scope of the EGM

Our intervention and outcomes framework was developed and adapted from the National Endowment for Science, Technology and the Arts (NESTA) and the FSC's Common Outcomes Framework (Future Skills Centre, 2019). Our 15 intervention categories are based on NESTA's nine components for an inclusive and responsive learning ecosystem (Orlik, 2018), which are highlighted as recommendations on how to plan policy for skills provision. The FSC Common Outcomes Framework is a set of measures that focus on employment‐related outcomes approaches both within Canada and internationally. We held a stakeholder workshop in February 2020 with academics, economists, and community‐based organizations in order to refine our interventions‐outcomes framework. Workshop participants suggested a recategorization of the framework categories and filter options, and these will be used in our map. The framework will follow a standard intervention and outcomes matrix where the rows are interventions and the columns are outcomes.

### 3.3 Conceptual framework of the EGM

Figure 1 is our conceptual framework in which the inputs lead to the intended outcomes. Interventions, services, or programs are dependent upon investments from governments and or related stakeholders, as well as research. Through the skills development interventions, individuals acquire the skills and training to optimize their potential in the labor market.

This conceptual framework includes skills development interventions which recognize and seek to overcome systemic barriers to equitable employment, such as lack of sufficient wrap‐around support, need for culturally responsive interventions and need for commitment and activation measures from employers to ultimately support a diverse workforce and promote community well‐being.

### 3.4 Eligibility criteria

We will include studies which assess the impact of interventions or programs with a component of skills development and/or training among individuals post high school age. For example, a jobs search assistance program without a skills development component will be excluded. Interventions may take place in any setting (e.g., community centre, workplace, educational institutions, etc.) in high income countries. We provide more details on our dimensions below.

### 3.5 Dimensions

The EGM framework for an EGM informs the inclusion and exclusion criteria. We chose intervention and outcomes as our key dimensions.

#### 3.5.1 Types of study designs

We will include primary studies and systematic reviews evaluating the effectiveness of interventions.

For primary studies, we will use the Maryland Scientific Methods Scale as it is adapted by the What works Centre for Local Economic Growth (Madaleno & Waights, 2014) which ranks impact evaluations by the robustness of the methodology of the studies and the quality of implementation. The scale provides a score from 1 to 5, where 1 is the least robust (e.g., cross‐sectional comparison of treated groups with untreated groups) and 5 is the most robust (e.g., randomized controlled trials). We included primary studies if the methods scored between levels 3 and 5. The included levels are defined as follows:

1. Level 3: outcomes are compared in intervention group before and after the intervention, and the comparison group is used to provide a counterfactual.
2. Level 4: the intervention and control groups vary in their exposure to the random allocation of the intervention.
3. Level 5: research designs that involve explicit randomization into intervention and control groups.

For systematic reviews, we will include studies that explicitly describe the methods used to identify studies (i.e., a search strategy), strategies for study selection (e.g., eligibility criteria and selection process) and methods of synthesis or analysis (Moher et al., 2015). This study design, as defined by PRISMA, uses a transparent and an a priori methodology in order to ensure rigor. We will not exclude eligible systematic reviews with level 1 or 2 studies.

We will list articles and books under studies awaiting classification if they are not available electronically due to the rapid process of this map.

#### 3.5.2 Treatment of qualitative research

We do not plan to include qualitative research due to the rapid nature of the map.

#### 3.5.3 Types of settings

We will limit primary studies to high income country settings as defined by the World Bank Classifications (The World Bank, 2020). We will not exclude systematic reviews that do not report the settings.

#### 3.5.4 Status of studies

We will include all relevant on‐going and completed studies. We will not search for unpublished articles.

#### 3.5.5 Population

Our map will focus on adults who are post high school age in high income countries. We recognize that individuals may drop out from school and the age at which individuals complete high school may vary between countries and provinces/states. For example, the typical age for completing high school is 18–19 years in Europe and 17–18 years in North America. We will also include studies with a subset of eligible participants as some studies may include participants with overlapping age range, for example.

Intervention: the intervention categories of this map, as outlined below, are adapted from the NESTA (Orlik, 2018) categorization for an inclusive and responsive skills ecosystem. Our interventions are listed and defined in Table 1.

**Table 1:** Intervention categories

Intervention	Definition
Career support and pathway guidance	Assistance to individuals who have been reskilled to enter a new sector and apply their skills, as well as help to find and choose high quality education and training that fits with their career aspirations and the needs of the labor market
	Examples: job search assistance, career guidance advice, resume support, pre‐employment services, digital job matching platforms
Assessment tools	Tools which help an individual get the right type of training to identify existing skills and competencies and areas that need improvement, as well as tools to determine individuals' skills or knowledge for placement, progression or certification in training programs
	Examples: studies that validate assessments of skills or learning
Funding—financial incentives and supports	Any form of financial incentives and/or supports to undertake and complete training for individuals and/or businesses
	Examples: vouchers, grants, training benefits
Wraparound support	Additional support and services that indirectly help and motivate individuals to complete any type of skills development or training
	Examples: childcare, coaching, counseling or therapy, networking, work with employers on job preparedness, postemployment support
Accreditation	Learners need to know which courses to trust, while employers want to know that candidates and employees are competent in the skills they claim to have third party recognition is often necessary to provide this assurance
	Examples: microcredentials, certificates, professional designations, certifying prior learning assessment
Culturally relevant and responsive interventions	Programs offering skills training need to explicitly recognize and build on cultural frames of reference to ensure participants experience respect, connection and a sense of personal and collective relevance
	Examples: Afrocentric or Indigenous cultural support, awareness and diversity training for all employees
Training delivery: literacy, numeracy, and additional language training	People in work need access to relevant, high quality training opportunities that fit their circumstances and preferred approaches to learning. Interventions listed in this category cover training programs focused on literacy, numeracy and additional language training
	Examples: job‐embedded basic skills programs, postsecondary access programs focused on literacy and/or numeracy, English as a second language training, actualisation linguistique en français
Training delivery: social‐emotional skills	People in work need access to relevant, high quality training opportunities that fit their circumstances and preferred approaches to learning. Interventions listed in this category cover training programs focused on social‐emotional skills
	Examples: 21st century skills, self‐awareness, self‐management, social awareness, relationship skills, responsible decision‐making, creativity, problem‐solving, interviewing skills
Training delivery: technical and occupation‐specific	People in work need access to relevant, high quality training opportunities that fit their circumstances and preferred approaches to learning. Interventions listed in this category cover technical and occupation‐specific training programs
	Examples: reskilling for displaced workers, upskilling for low‐skill workers
Training delivery: digital skills	People in work need access to relevant, high quality training opportunities that fit their circumstances and preferred approaches to learning. Interventions listed in this category cover training programs focused on digital skills
	Examples: introduction to digital technologies, skills for the digital age
Training delivery: work experience‐based learning	People in work need access to relevant, high quality training opportunities that fit their circumstances and preferred approaches to learning. Interventions listed in this category covers work experience‐based learning
	Examples: apprenticeship, internship, work‐integrated learning
Activation or commitment measures	Governments or institutions may set targets or make commitments to promote skills development or access to workforce from historically marginalized groups
	Examples: national youth guarantee programs (Finland Youth), equity targets, community benefit agreements
Consortium‐based models	In order to deliver training and workforce development services, organizations representing different institutions or systems work together
	Examples: Sector‐based, school board‐college partnerships, partnerships between educational institutions and employers, regionally based partnerships
Skills utilization practices by employers	Work organization and management practices that reward and facilitate the application of new skills, complement skill development, and amplify the returns to learning
	Examples: high performance work practices, job redesign, skills audits, multiskilling, job rotation
Employer recruitment approaches	Interventions that foment change in the way employers hire, in order to address skills gaps
	Examples: anti bias training, blinded hiring processes

We will include studies with interventions that have a component of skills development and/or training and exclude all studies which do not have a skills development and/or training component. For example, this exclusion may include the WorkFirst program, Individual placement and support, or subsidized entrepreneurship if there is no focus on skills or training.

#### 3.5.6 Outcomes

We adapted the Common Outcomes Framework from the FSC (Future Skills Centre, 2019). The outcomes we will map are found below in Table 2.

**Table 2:** Outcome categories

Outcome category	
Jobseeker/worker outcomes	Program completion: successfully completing planned activities
	Learner motivation
	Search duration: time between unemployment and employment
	Skills gains: measured gains in specific skills
	Credential attainment: attainment of high school or PSE credentials, field of study of credentials
	Employment and retention: employment status, nature of employment (permanent, temporary, full/part‐time), retention
	Earnings: hours worked per week, wages, annual earnings
	Benefits: paid leave, health and dental coverage, pension plan
	Job security
	Enrollment in further education
	Well‐being: quality of life
	Civic engagement
	Adverse effects: any unintended outcomes, harms
Employer outcomes	Program satisfaction: satisfaction with program, perceived utility of program, likelihood to recommend
	Hire quality: satisfaction with hires through the program, perceived skill of program hires relative to other channels, perceived fit of program hires relative to other channels, perception of hiring needs being met
	Hiring channel satisfaction: satisfaction with hiring channel, perceived ease of hiring process relative to other channels
	Retention: retention of employees hired through the program, earnings trajectory of employees hired through the program
System‐level outcomes	Diversity of workforce/equity
	Community well‐being

### 3.6 Search methods and sources

We will develop and pilot a search strategy (with a selection of suggested studies that met our inclusion criteria) with an information scientist (Douglas Salzwedel). This search will comprise social and economic databases including gray literature sources such as dissertations and conference abstracts databases. We will use the search strategy for on‐going and completed studies in: ABI/INFORM via ProQuest, Canadian Business & Current Affairs Database via ProQuest, Canadian Research Index via ProQuest, EconLit via EBSCO, ERIC via ProQuest, International Bibliography of the Social Sciences via ProQuest, ProQuest Dissertations & Theses Global, PsycINFO via Ovid, Social Services Abstracts via ProQuest, Sociological Abstracts via ProQuest, Web of Science via Clarivate (Social Sciences Citation Index and Conference Proceedings Citation Index—Social Science & Humanities), and Social Systems Evidence. See Table 3 for a full PsycInfo search strategy. We held a stakeholder meeting with FSC to invite suggestions of relevant studies and sources. We will not contact other organizations or individuals for information about unpublished or ongoing studies.

**Table 3:** Search strategy in Psychinfo

Database: APA PsycInfo <1806 to March Week 2 2020> via Ovid
Search date: 15 March, 2020
1. employee skills/(1404) 2. supported employment/(1366) 3. ((accreditation or advisor* or assessment* or assist* or coach* or counsel* or develop* or financ* or guidance or incentiv* or innovat* or intervention* or mentor* or partnership* or placement* or policies or policy or project or projects or program or programme or programmes or programs or promot* or scheme or subsidies or subsidised or subsidized or subsidy or support*) adj2 (career* or course or courses or employment or entrepreneur* or job or jobs or learn* or retrain* or skill or skills or train or training or upgrade* or upgrading or upskill*)).ti. (25645) 4. or/1‐3 (27687) 5. job search/(1386) 6. job applicants/(1496) 7. employment status/(15374) 8. unemployment/(4204) 9. occupational guidance/(8526) 10. vocational rehabilitation/(5943) 11. supported employment/(1366) 12. (adult* or applicant* or claimant* or graduat* or immigrant* or laborer* or labourer* or mature learner* or men or migrant* or native* or newcomer* or participant* or “post‐secondary” or recipient* or unemploy* or women or worker* or young* or youth*).ti. (335512) 13. or/5‐12 (362215) 14. “systematic review”/(286) 15. Meta Analysis/(4629) 16. Randomized Controlled Trials/(318) 17. Quasi Experimental Methods/(183) 18. (allocat* or assign* or control group* or controlled or econometric* or evidence review or metaanalysis or meta‐analysis or microeconometric* or propensity score or quasi‐experiment* or quasi experiment* or randomi* or randomly or synthesi* or trial or trials or systematic literature review or systematic review or treatment* or ((experiment or experimental) adj2 (design* or evaluat* or evidence or research or studies or study)) or (regress* adj3 discontin*) or ((assess* or effect* or evaluat*) adj4 (impact* or program*)) or (difference* adj3 difference*) or (match* adj2 (coarsened or covariate or propensity)) or ((counterfactual or estimat*) and evaluat*) or ((“instrumental variable” or IV) adj3 (estimat* or approach))).ti,ab. (1564305) 19. or/14‐18 (1564913) 20. and 13 and 19 (1910) 21. 21 limit 20 to yr = “2000 ‐ 2020” (1344)

### 3.7 Screening and selection of studies

We adhered to the Cochrane Rapid Review Guidance (Garritty et al., 2020) as this present map is a rapid EGM. We will use two reviewers for dual screening of at least 20% of abstracts, with conflict resolution. We will use single screening for the remaining abstracts and have a second screener to screen all excluded titles and resolve any conflicts. Title and abstracts will be screened based on intervention, study design (i.e., levels on the Maryland Scientific Methods Scale), population, year of publication, language, and not based on outcome. We will exclude studies published before 2000, level 1 or 2 studies and studies that are not in either English or French. We will use Rayyan for screening at this stage and utilize the program's text‐mining function to calculate likelihood of inclusion (Ouzzani et al., 2016). For full‐text screening, we will have dual screening for approximately 20% of studies, and have conflicts resolved by discussion or a third reviewer. The remaining studies will be screened by one reviewer, and a second reviewer will screen excluded studies. We will use EPPI‐Reviewer to screen at the full‐text stage. We will also screen the reference lists of relevant studies and systematic reviews identified. We will not contact authors of studies or reviews for missing information.

### 3.8 Data extraction, coding, and management

Included studies will be coded by a single reviewer and we will check for consistency and agreement on ~20% of studies. Our coding categories for data extraction are based on our framework. We will also collect details important for our stakeholders such as region (i.e., Canada, United States, Europe, East Asia and Pacific, Latin America and the Caribbean, other), setting (i.e., urban or rural), population focus for intervention (e.g., francophone, newcomers, low‐income, disability or Indigenous populations), age group (i.e., 15–24, 25–54, 55–64, 65+ years), auspices (e.g., employment services, digital delivery), and sociodemographics (e.g., education, marital status). These will be included as filters in the map. We have piloted and tested the extraction form on a sample of studies and generated a draft map to discuss with our stakeholders. Refer to Table 4 for coding categories.

**Table 4:** Coding categories

Category	Sub‐categories (as applicable)
Publication status	1. Complete 2. On‐going
Study design	1. Primary study 2. Systematic review
Primary study design	1. Level 3 2. Level 4 3. Level 5
Region	1. Canada 2. United States 3. Europe 4. East Asia and Pacific 5. Latin America and the Caribbean 6. Other
Canada	1. British Columbia 2. Alberta 3. Saskatchewan 4. Manitoba 5. Ontario 6. Quebec 7. Nova Scotia 8. Prince Edward Island 9. Newfoundland and Labrador 10. New Brunswick 11. Northwest Territories 12. Yukon 13. Nunavut
Setting	1. Rural 2. Urban
Population focus for intervention	1. Francophone 2. Other linguistic minorities 3. LGBTQ+ 4. Men 5. Women 6. Newcomers 7. Disability (all) 8. Indigenous populations 9. Racialized status (any group which faces discrimination based on their race/ethnicity/culture) 10. Low skill 11. Low‐income 12. 15–24 (youth) 13. 25–54 (young adults/adults) 14. 55–64 (adults) 15. 65+ (older adults) 16. Individuals on medical leave (e.g., disability leave, sick leave) 17. Individuals on other leave (e.g., maternity leave, sabbatical, etc.)
Age group	1. 15–24 2. 25–54 3. 55–64 4. 65+
Interventions	1. Career support and pathway guidance 2. Assessment tools 3. Funding—financial incentives and supports 4. Wraparound support 5. Accreditation 6. Culturally relevant and responsive interventions 7. Training delivery: literacy, numeracy and additional language training 8. Training delivery: social‐emotional skills 9. Training delivery: technical and occupation‐specific 10. Training ddelivery: digital skills 11. Training delivery: work experience‐based learning 12. Activation or commitment measures 13. Consortium‐based models 14. Skills utilization practices by employers 15. Employer recruitment approaches
	Jobseeker/worker outcomes
	1. Program completion 2. Learner motivation 3. Search duration 4. Skills gains 5. Credential attainment 6. Employment and retention 7. Earnings 8. Benefits 9. Job security 10. Enrollment in further education 11. Well‐being 12. Civic engagement 13. Adverse effects
	Employer outcomes
	1. Program satisfaction 2. Hire quality 3. Hiring channel satisfaction 4. Retention
	System‐level outcomes
	1. Diversity of workforce/equity 2. Community well‐being
Socio‐demographic filters	1. Gender/sex 2. LGBTQ+ 3. Marital status 4. Education 5. Household composition (e.g., children in household) 6. Immigration status 7. Disability 8. Ethnicity/race
Auspices:	1. University/degree 2. College/diploma certificate 3. Apprenticeship 4. Community‐based 5. Private career institutes 6. Employment services 7. Regulated trade or professional 8. Sub‐diploma/microcredential 9. Digital (online, virtual) delivery

### 3.9 Quality appraisal

We will appraise the methodological quality of systematic reviews with AMSTAR‐2 (Shea et al., 2017) in duplicate for 20% of eligible studies. *κ* statistics will also be used to check agreement for each item. If the agreement is over 80%, we will proceed with single data extraction with verification by a second reviewer for the remainder of studies. The methodological quality of primary studies is not usually assessed in EGMs (Snilstveit et al., 2017).

## 4 ANALYSIS AND PRESENTATION

### 4.1 Report structure

Our EGM report will include the standard sections: executive summary, background, methods, results, and conclusion. We will indicate any changes we made between the protocol and the final report. The results section will present the number of studies retrieved from our database search and provide an overview of the types of studies by intervention, outcomes, auspices and other filters used. The conclusion will provide a detailed account of policy implications for researchers and decision‐makers and highlight key areas for research commissioning.

We will include the following tables and figures:

1. Figure: Conceptual framework
2. Figure: PRISMA flow chart
3. Table: number of studies by study design
4. Table: number of studies by interventions and outcomes
5. Table: number of studies by population
6. Other tables and figures will be included based on the coded information for other filters.

#### 4.1.1 Dependency

We will treat multiple reports (e.g., secondary analyses or protocols) of a single study as one. Systematic reviews will likely include primary studies that are included in the map, and there may be more than one systematic review which may include the same primary study. Primary studies which meet our eligibility criteria will be included in the map regardless of whether they are included in a systematic review.

#### 4.1.2 The evidence gap map

The key dimensions will be interventions (presented in rows) and outcomes (presented in columns). We will use bubbles of varying sizes to present included studies. Different colors will be used for different types of studies. Final decisions about the filters will be made based on the number of included studies and coded information. The online interactive map will be hosted by FSC and Campbell.

## 5 STAKEHOLDER ENGAGEMENT

Our central team (V. W., K. G., L. M., and C. M.) will meet bi‐weekly to discuss the direction and scope of the EGM. We held a stakeholder workshop on February 26th, 2020 with our advisory group to discuss our proposed intervention and outcome framework, and eligible study designs. This group of stakeholders represented perspectives from community‐based organizations as well as academics and economists who work in the skills development and training sector in Canada. The advisory group will convene again in May to discuss a draft map with preliminary results.

## ROLES AND RESPONSIBILITIES

Content: L. M. M., K. and G.‐M.

EGM methods: C. M. and V. W.

Information retrieval: Douglas Salzwedel (consultant).

## SOURCES OF SUPPORT

This EGM is supported by the Future Skills Centre of Canada.

## DECLARATIONS OF INTEREST

Luciana Manhaes Marins and Kelly Gallagher‐Mackay work at Future Skills Centre in Canada and intend to use this map to discuss priority action and research areas with stakeholders.

## PRELIMINARY TIMEFRAME

Approximate date for submission of the EGM: June 2020

## PLANS FOR UPDATING THE EGM

This EGM will be updated every 2 years.
